# Long non‐coding RNA MALAT1 and its target microRNA‐125b associate with disease risk, severity, and major adverse cardiovascular event of coronary heart disease

**DOI:** 10.1002/jcla.23593

**Published:** 2021-03-04

**Authors:** Fanqin Lv, Liang Liu, Qiang Feng, Xuefeng Yang

**Affiliations:** ^1^ Department of Cardiology Daqing Fifth Hospital Daqing China; ^2^ Department of Cardiology Daqing Oil Field General Hospital Daqing China; ^3^ Department of Cardiology HanDan Central Hospital HanDan China; ^4^ Department of Thoracic Surgery Daqing Oil Field General Hospital Daqing China

**Keywords:** coronary heart disease, Gensini score, long non‐coding RNA MALAT1, major adverse cardiovascular event, microRNA 125b

## Abstract

**Background:**

This study aimed to explore the correlation of long non‐coding RNA metastasis‐associated lung adenocarcinoma transcript 1 (lncRNA MALAT1) with microRNA (miR)‐125b and further investigated their associations with disease risk, severity, and prognosis of coronary heart disease (CHD).

**Methods:**

Totally, 230 patients who underwent diagnostic coronary angiography were recruited; meanwhile, 140 of them were diagnosed as CHD and the remaining 90 non‐CHD patients served as controls. Plasma sample was collected from each participant for lncRNA MALAT1 and miR‐125b mRNA expression detection by reverse transcription‐quantitative polymerase chain reaction. The extent of coronary stenosis was evaluated by the Gensini score, and major adverse cardiovascular event (MACE) occurrence during the follow‐up was documented in CHD patients.

**Results:**

Long non‐coding RNA metastasis‐associated lung adenocarcinoma transcript 1 relative expression was increased, but miR‐125b relative expression was decreased in CHD patients compared with controls. ROC curve exhibited that lncRNA MALAT1 and miR‐125b were of good value in differentiating CHD patients from controls, and further logistic regression analysis verified their independent correlation with CHD risk. Furthermore, lncRNA MALAT1 presented a closely negative correlation with miR‐125b in CHD patients, while it presented a weakly negative association with miR‐125b in controls. In CHD patients, lncRNA MALAT1 was positively correlated with Gensini score, total cholesterol, low‐density lipoprotein cholesterol, C‐reactive protein, tumor necrosis factor α, interleukin (IL)‐1β, IL‐6, IL‐17, and accumulating MACE occurrence; reversely, miR‐125b presented a opposite trend.

**Conclusion:**

Long non‐coding RNA metastasis‐associated lung adenocarcinoma transcript 1 might be associated with increased CHD risk, severity, and accumulating MACE incidence via negative interaction with miR‐125b, suggesting their possible clinical application as biomarkers in the CHD screening and surveillance.

## Graphical abstract

Totally, 230 patients who underwent diagnostic coronary angiography were recruited; meanwhile, 140 of them were diagnosed as CHD patients and the remaining 90 non‐CHD patients served as controls. Plasma sample was collected from each participant for lncRNA MALAT1 and miR‐125b mRNA expression detection by reverse transcription‐quantitative polymerase chain reaction. The extent of coronary stenosis was evaluated by the Gensini score, and major adverse cardiovascular event (MACE) occurrence during the follow‐up was documented in CHD patients. Results indicated that long non‐coding RNA metastasis‐associated lung adenocarcinoma transcript 1 relative expression was increased, but miR‐125b relative expression was decreased in CHD patients compared with controls. ROC curve exhibited that lncRNA MALAT1 and miR‐125b were of good value in differentiating CHD patients from controls, and further logistic regression analysis verified their independent correlation with CHD risk. Furthermore, lncRNA MALAT1 presented a closely negative correlation with miR‐125b in CHD patients, while it presented a weakly negative association with miR‐125b in controls. In CHD patients, lncRNA MALAT1 was positively correlated with Gensini score, total cholesterol, low‐density lipoprotein cholesterol, C‐reactive protein, tumor necrosis factor‐α, interleukin (IL)‐1β, IL‐6, IL‐17, and accumulating MACE occurrence; reversely, miR‐125b presented an opposite trend.

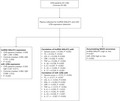

## 1 INTRODUCTION

Coronary heart disease (CHD) remains the leading cause of morbidity and mortality globally, and the death rate of CHD has experienced a rise in the last decades considering the prevalence of obesity and lifestyle changes.^1^, ^2^ According to the global epidemiological data, it is estimated that there are approximately 93 million people suffering from CHD, which eventually contributes to 8.1 million deaths.^3^ Pathologically, CHD is considered to be caused by atherosclerosis‐induced arterial stenosis, and recent papers indicate that inflammation also plays an important role in the formation of atherosclerotic plaque, further leading to arterial stenosis.^4^, ^5^ Current clinical management, including arterial revascularization (such as percutaneous coronary intervention and coronary artery bypass grafting) and drug treatments (such as aspirin), represents great therapeutic advance for CHD patients; however, a portion of patients still suffer from unfavorable prognosis as the result of reoccurred coronary events.^5^, ^6^

Long non‐coding RNA metastasis‐associated lung adenocarcinoma transcript 1 (lncRNA MALAT1) is recognized as a biomarker in various cancers, and recent studies indicate that it is also involved in the pathogenesis of atherosclerosis.^7^, ^8^, ^9^, ^10^ Mechanically, lncRNA MALAT1 silencing attenuates oxidized low‐density lipoprotein (ox‐LDL)‐induced endothelial inflammation and protects the endothelium from oxidative stress, further affecting the progression of atherosclerosis.^7^ Additionally, another study indicates that lncRNA MALAT1 affects lipid metabolism disorder and is associated with inflammation response as well as the pathological progression of atherosclerosis via regulating ox‐LDL‐related macrophages.^8^ Furthermore, lncRNA MALAT1 is reported to regulate cardiac inflammation and promote acute myocardial infarction via interaction of microRNA‐125b (miR‐125b), and miR‐125b serves as a pathogenic mediator in multiple vascular diseases.^11^, ^12^, ^13^ For example, miR‐125b attenuates endothelin‐1 expression, suppresses vascular endothelial‐cadherin mRNA translation, and reduces endothelial permeability, involving the development of atherosclerosis.^13^, ^14^ Furthermore, the preliminary experiments of our study observed that lncRNA MALAT1 was upregulated but miR‐125b was downregulated in CHD patients compared with controls. According to the aforementioned evidence and considering that the occurrence of CHD commonly had the pathophysiological basis of atherosclerosis accompanying with chronic inflammation and high lipid level, we hypothesized that lncRNA MALAT1 might be correlated with higher CHD risk, and presented correlation with increased level of coronary artery stenosis, lipid profile, and inflammation, contributing to enhanced severity and poor prognosis via interaction with miR‐125b; however, to our best knowledge, there was no related research until now.^15^, ^16^, ^17^

In the present study, we detected the correlation of lncRNA MALAT1 and miR‐125b, and further investigated their potential in predicting CHD risk as well as their correlation with disease severity, level of lipid profile as well as inflammatory cytokines, and major adverse cardiovascular event (MACE) occurrence in CHD patients.

## 2 MATERIALS AND METHODS

### 2.1 Subjects

From February 2015 to September 2016, 230 patients were recruited in this study when they underwent diagnostic coronary angiography in our hospital due to unexplained chest pain or suspected CHD symptoms (such as angina, chest oppression, and short of breath). All recruited patients were older than 18 years and required to be in the absence of cardiomyopathy, congenital heart disease, severe liver or renal diseases, severe infection, sepsis, systemic autoimmune diseases (eg, rheumatoid arthritis), malignant hematological diseases, or tumors, and have no history of cardiovascular surgery (eg, coronary artery bypass graft surgery or percutaneous transluminal coronary angioplasty). The pregnant or lactating women were not included in the study. Through diagnostic coronary angiography, 140 of the 230 patients were diagnosed as CHD based on there was at least one major epicardial vessel with >50% stenosis and presenting with typical angina. The remaining 90 non‐CHD patients were severed as control subjects in the analysis. This study was approved by the Institutional Review Board of our hospital. All participants provided the written informed consent.

### 2.2 Clinical data collection

Clinical characteristics of patients were collected by interview before coronary angiography, including demographics (age, gender, and body mass index), smoke status, and medical histories (family history of CHD, hypertension, hyperlipidemia, hyperuricemia, and diabetes mellitus [DM]). Following the laboratory tests, the biochemical indexes were recorded, such as fasting blood glucose (FBG), serum creatinine, serum uric acid, triglyceride (TG), total cholesterol (TC), LDL cholesterol (LDL‐C), high‐density lipoprotein cholesterol (HDL‐C), and C‐reactive protein (CRP). Additionally, by coronary angiography, the extent of coronary stenosis was evaluated by the Gensini score according to the previous study,^18^ where a higher score indicated more severe coronary stenosis.

### 2.3 Peripheral blood collection and detection

Peripheral blood samples of CHD patients and non‐CHD patients (as controls) were extracted using anticoagulant tube before coronary angiography. After collection, the PB sample was centrifuged at 4°C and 1600 *g* for 10 minutes immediately; then, plasma was collected. Following that, the plasma was further centrifuged at 4°C and 16 000 *g* for 15 minutes to completely remove the cell debris. Thereafter, the lncRNA MALAT1 and miR‐125b expressions in plasma of CHD patients and controls were determined by reverse transcription‐quantitative polymerase chain reaction (RT‐qPCR). For further analysis, the inflammatory cytokine (tumor necrosis factor α [TNF‐α], interleukin 1β [IL‐1β], IL‐6, IL‐8, IL‐10, and IL‐17) levels in the plasma of CHD patients were measured using human enzyme‐linked immunosorbent assay kits (Abcam) according to the manufacturer's handbook.

### 2.4 RT‐qPCR assay

The level of lncRNA MALAT1 and miR‐125b in plasma was detected by RT‐qPCR for all participants included. RNA extraction was conducted using QIAamp RNA Blood Mini Kit (Qiagen); subsequently, reverse transcription was performed with iScript™ cDNA Synthesis Kit (Bio‐Rad) according to the manufacturer's instruction. qPCR was performed with SYBR^®^ Green Realtime PCR Master Mix (Toyobo) according to the manufacturer's guidance. lncRNA MALAT1 and miR‐125b were detected via qPCR, and qPCRs were performed in triplicate with lncRNA MALAT1 internal coefficient variation of 1.8% in CHD patients and 1.2% in controls as well as with miR‐125b internal coefficient variation of 0.8% in CHD patients and 1.5% in controls. The relative expressions of lncRNA MALAT1 and miR‐125b were calculated using the formula of 2^−ΔΔCt^, with Glyceraldehyde‐3‐phosphate dehydrogenase (GAPDH) (for lncRNA MALAT1) and U6 (for miR‐125b) used as the internal references. Detailed calculation process was as follows: (a) qPCR was performed in triplicate, and the average of lncRNA MALAT1 Ct, miR‐125b Ct, GAPDH Ct, and U6 Ct in every sample was determined, respectively. (b) Calculations of ΔCt (Ct_avg. lncRNA MALAT1_ − Ct_avg. GAPDH_) and ΔCt (Ct_avg. miR‐125b_ − Ct_avg. U6_) were presented in every sample, which were shown as ΔCt_(sample)_. (c) The median of ΔCt in controls was referred as the calibrator, which was shown as ΔCt_(calibrator)_. (d) ΔΔCt = ΔCt_(sample)_ − ΔCt _(calibrator)_. (e) The relative expressions of lncRNA MALAT1 and miR‐125b were proceeded via calculating 2^−ΔΔCt^, respectively. The primers were as follows: lncRNA MALAT1, forward (5ʹ‐>3ʹ): TCCTAAGGTCAAGAGAAGTGTCAG, reverse (5ʹ‐>3ʹ): GTGGCGATGTGGCAGAGAA; miR‐125b, forward (5ʹ‐>3ʹ): ACACTCCAGCTGGGTCCCTGAGACCCTAACTT, reverse (5ʹ‐>3ʹ): TGTCGTGGAGTCGGCAATTC; GAPDH, forward (5ʹ‐>3ʹ): TGACCACAGTCCATGCCATCAC, reverse (5ʹ‐>3ʹ): GCCTGCTTCACCACCTTCTTGA; and U6, forward (5ʹ‐>3ʹ): CTCGCTTCGGCAGCACATATACTA, reverse (5ʹ‐>3ʹ): ACGAATTTGCGTGTCATCCTTGC.

### 2.5 MACE assessment

Major adverse cardiovascular event occurrence during the follow‐up was documented, which was defined as the composite of cardiovascular death, acute coronary syndrome, unscheduled revascularization, or hospital admission for cardiovascular cause.^19^

All CHD patients were continuously followed up to 36 months by telephone contacts or clinic visits. Accumulating MACE occurrence was evaluated from the date of enrollment to the date of the MACE occurrence. A total of 18 patients were lost to follow‐up during the follow‐up period, and those patients were censored on the date of the last visit when analyzing the accumulating MACE occurrence.

### 2.6 Statistical analysis

SPSS 22.0 statistical software (IBM) was used for data analysis, and GraphPad Prism 7.01 (GraphPad Software Inc) was used for figure plotting. Continuous variables were described as mean with standard deviation (SD), or median with interquartile range (IQR). Categorized variables were expressed as count (percentage). Student's *t* test or Wilcoxon rank‐sum test was performed to determine the difference of continuous data between two groups; chi‐square test was performed to determine the difference of categorized data between two groups; Spearman's rank correlation test was performed to determine the correlation between two continuous variables. Receiver operating characteristic (ROC) curves with area under the ROC curve (AUC) were used to illustrate performance of variables in distinguishing different subjects. In order to analyze the correlation of lncRNA MALAT1 and miR‐125b with accumulating MACE occurrence, lncRNA MALAT1 and miR‐125b were further categorized as high expression and low expression, respectively, based on their median values. Kaplan‐Meier method was used to display accumulating MACE occurrence, and the log‐rank test was carried out to determine the difference of accumulating MACE occurrence between two groups. Univariate and forward stepwise multivariate logistic regressions were performed to analyze the factors associated with CHD risk. Univariate and forward stepwise multivariate Cox's proportional hazard regressions were conducted to analyze the factors predicting accumulating MACE occurrence. *P* < .05 was considered as statistically significant.

## 3 RESULTS

### 3.1 Comparison of clinical characteristics between CHD patients and controls

The mean age was 62.6 ± 9.8 years for CHD patients and 58.3 ± 9.6 years for controls, respectively (Table 1). There were 25 (17.9%) females and 115 (82.1%) males in CHD patients, and there were 21 (23.3%) females and 69 (76.7%) males in controls. CHD patients presented increased age (*P* = .001), hypertension (*P* = .036), FBG (*P* = .034), CRP (*P* < .001), and Gensini score (*P* < .001) but decreased HDL‐C (*P* = .006) compared with controls. No difference of other clinical characteristics was shown between CHD patients and controls (all *P* > .05). More detailed information of clinical characteristics is shown in Table 1.

**TABLE 1:** Clinical characteristics

Items	Controls (N = 90)	CHD patients (N = 140)	*P* value
Age (y), mean ± SD	58.3 ± 9.6	62.6 ± 9.8	.001
Gender, no. (%)			.311
Female	21 (23.3)	25 (17.9)	
Male	69 (76.7)	115 (82.1)	
BMI (kg/m^2^), mean ± SD	23.4 ± 3.1	24.0 ± 2.8	.110
Hypertension, no. (%)	58 (64.4)	108 (77.1)	.036
Hyperlipidemia, no. (%)	40 (44.4)	73 (52.1)	.254
Hyperuricemia, no. (%)	30 (33.3)	54 (38.6)	.421
DM, no. (%)	13 (14.4)	35 (25.0)	.055
Smoke, no. (%)	32 (35.6)	62 (44.3)	.189
Family history of CHD, no. (%)	16 (17.8)	30 (21.4)	.499
FBG (mmol/L), median (IQR)	5.3 (4.8‐6.1)	5.7 (5.0‐6.5)	.034
Scr (μmol/L), median (IQR)	74.0 (65.9‐85.6)	77.4 (64.9‐84.6)	.485
SUA (μmol/L), median (IQR)	364.7 (319.6‐401.3)	352.3 (312.9‐401.9)	.538
TG (mmol/L), median (IQR)	1.4 (0.9‐2.3)	1.7 (0.9‐2.3)	.265
TC (mmol/L), median (IQR)	4.7 (3.7‐5.4)	4.7 (4.1‐5.5)	.192
LDL‐C (mmol/L), median (IQR)	2.9 (2.4‐3.3)	2.9 (2.6‐3.4)	.153
HDL‐C (mmol/L), median (IQR)	1.0 (0.8‐1.2)	0.9 (0.8‐1.1)	.006
CRP (mg/L), median (IQR)	7.0 (2.9‐10.2)	11.7 (8.6‐14.9)	<.001
Gensini score, median (IQR)	1.0 (0.0‐2.0)	41.0 (22.5‐58.5)	<.001

Abbreviations: BMI, body mass index; CHD, coronary heart disease; CRP, C‐reactive protein; DM, diabetes mellitus; FBG, fasting blood glucose; HDL‐C, high‐density lipoprotein cholesterol; IQR, interquartile range; LDL‐C, low‐density lipoprotein cholesterol; Scr, serum creatinine; SD, standard deviation; SUA, serum uric acid; TC, total cholesterol; TG, triglyceride.

### 3.2 Correlation of lncRNA MALAT1 and miR‐125b with CHD risk

**FIGURE 1:**
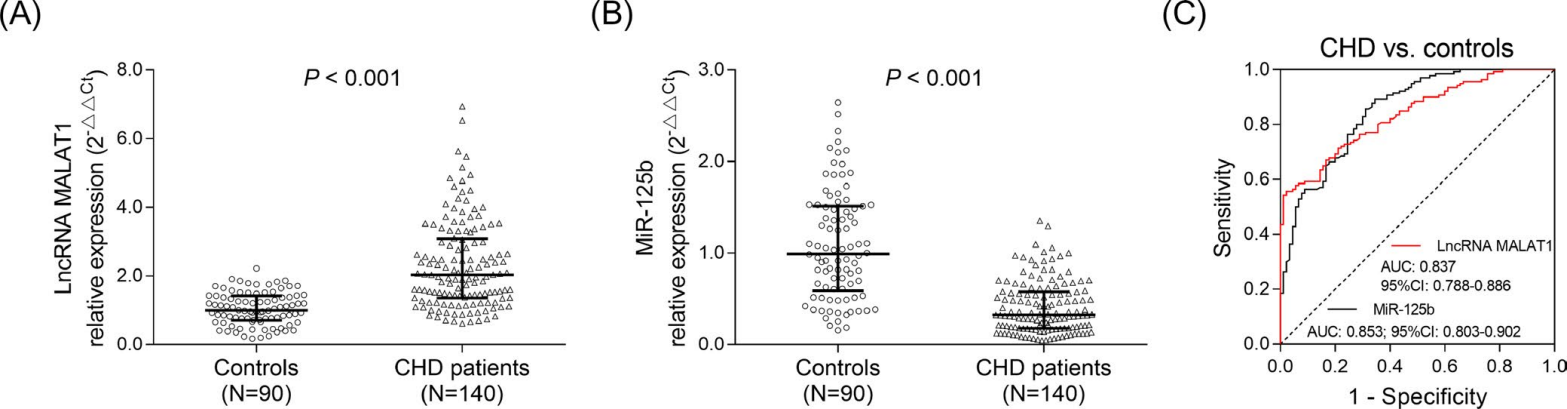
lncRNA MALAT1 and miR‐125b level in CHD patients and controls. Comparison of lncRNA MALAT1 (A)/miR‐125b (B) between CHD patients and controls. The ability of lncRNA MALAT1 and miR‐125b in differentiating CHD patients from controls (C). CHD, coronary heart disease; lncRNA MALAT1, long non‐coding RNA metastasis‐associated lung adenocarcinoma transcript 1; miR‐125b, microRNA 125b

Long non‐coding RNA metastasis‐associated lung adenocarcinoma transcript 1 relative expression was increased in CHD patients (median: 2.039; IQR: 1.364‐3.086) compared with controls (median: 0.995; IQR: 0.707‐1.426) (*P* < .001) (Figure 1A), while miR‐125b relative expression was decreased in CHD patients (median: 0.326; IQR: 0.197‐0.577) compared with controls (median: 0.993; IQR: 0.589‐1.515) (*P* < .001) (Figure 1B). ROC curve indicated that lncRNA MALAT1 (AUC: 0.837; 95% CI: 0.788‐0.886) and miR‐125b (AUC: 0.853; 95% CI: 0.803‐0.902) were both of good value in differentiating CHD patients from controls (Figure 1C).

### 3.3 Factors associated with CHD risk

Univariate logistic regression analysis indicated that lncRNA MALAT1 (OR = 8.409, *P* < .001), age (OR = 1.047,*P* = .001), hypertension (OR = 1.862, *P* = .037), FBG (OR = 1.318, *P* = .037), and CRP (OR = 1.219, *P* < .001) were positively correlated with CHD risk, while miR‐125b (OR = 0.028, *P* < .001) and HDL‐C (OR = 0.222, *P* = .006) were negatively associated with CHD risk (Table 2). Further forward stepwise multivariate logistic regression analysis revealed that lncRNA MALAT1 (OR = 4.182, *P* < .001), age (OR = 1.068, *P* = .004), hypertension (OR = 3.097, *P* = .018), DM (OR = 3.694, *P* = .029), and CRP (OR = 1.124, *P* = .007) were independent risk factor for CHD, but miR‐125b (OR = 0.053, *P* < .001) was an independent protective factor for CHD (Table 2).

**TABLE 2:** Factors associated with CHD risk

Items	Logistic regression model			
	*P* value	OR	95% CI	
			Lower	Higher
Univariate logistic regression				
lncRNA MALAT1	<.001	8.409	4.436	15.942
MiR‐125b	<.001	0.028	0.011	0.074
Age	.001	1.047	1.018	1.077
Male	.312	1.400	0.729	2.688
BMI	.111	1.077	0.983	1.181
Hypertension	.037	1.862	1.038	3.341
Hyperlipidemia	.255	1.362	0.800	2.318
Hyperuricemia	.421	1.256	0.721	2.187
DM	.057	1.974	0.979	3.981
Smoke	.189	1.441	0.835	2.486
Family history of CHD	.500	1.261	0.643	2.476
FBG	.037	1.318	1.017	1.709
Scr	.317	1.010	0.991	1.029
SUA	.871	1.000	0.995	1.004
TG	.225	1.239	0.876	1.752
TC	.109	1.238	0.953	1.608
LDL‐C	.071	1.424	0.970	2.090
HDL‐C	.006	0.222	0.075	0.652
CRP	<.001	1.219	1.138	1.306
Forward stepwise multivariate logistic regression				
lncRNA MALAT1	<.001	4.182	1.915	9.133
MiR‐125b	<.001	0.053	0.016	0.177
Age	.004	1.068	1.021	1.117
Hypertension	.018	3.097	1.215	7.890
DM	.029	3.694	1.146	11.906
CRP	.007	1.124	1.033	1.222

Abbreviations: BMI, body mass index; CI, confidence interval; CHD, coronary heart disease; CRP, C‐reactive protein; DM, diabetes mellitus; FBG, fasting blood glucose; HDL‐C, high‐density lipoprotein cholesterol; LDL‐C, low‐density lipoprotein cholesterol; lncRNA MALAT1, long non‐coding RNA metastasis‐associated lung adenocarcinoma transcript 1; miR‐125b, microRNA‐125b; OR, odds ratio; Scr, serum creatinine; SUA, serum uric acid; TG, triglyceride; TC, total cholesterol.

### 3.4 Correlation of lncRNA MALAT1 with miR‐125b in CHD patients and controls

**FIGURE 2:**
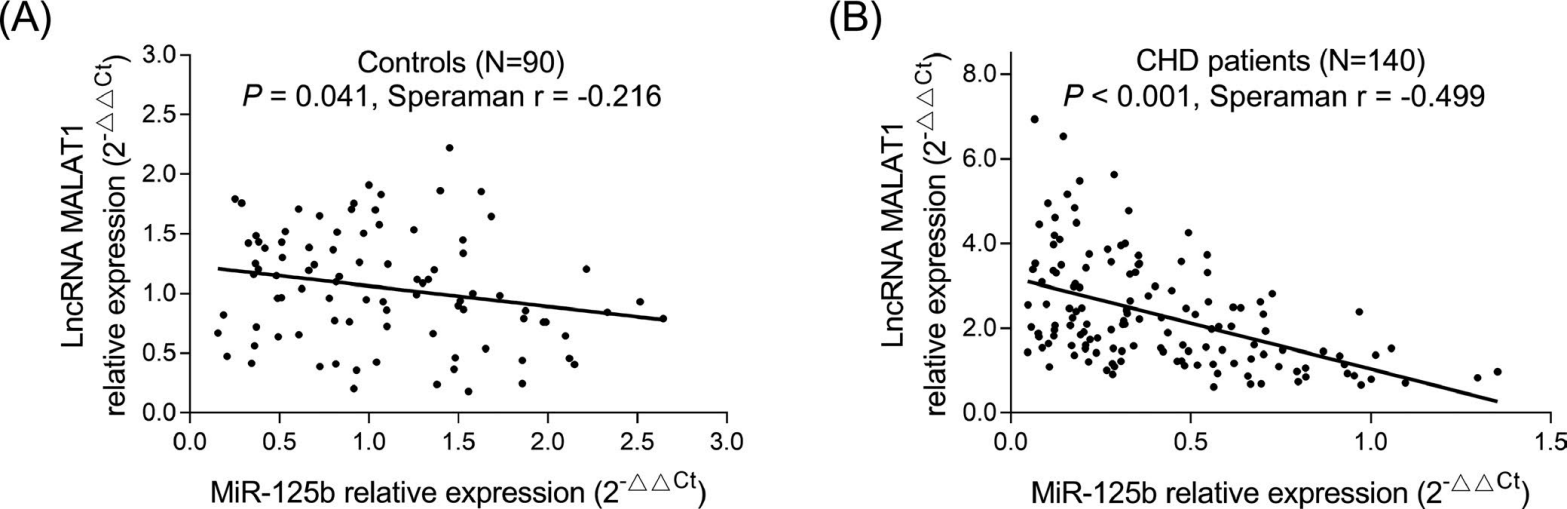
Association of lncRNA MALAT1 with miR‐125b in participants. Association of lncRNA MALAT1 with miR‐125b in controls (A) and CHD patients (B). CHD, coronary heart disease; lncRNA MALAT1, long non‐coding RNA metastasis‐associated lung adenocarcinoma transcript 1; miR‐125b, microRNA 125b

In controls, lncRNA MALAT1 was negatively associated with miR‐125b, but the correlation coefficient was low (*r* = −0.216, *P* = .041) (Figure 2A). Furthermore, in CHD patients, lncRNA MALAT1 showed a closely negative correlation with miR‐125b (*r* = −0.499, *P* < .001) (Figure 2B).

### 3.5 Correlation of lncRNA MALAT1 and miR‐125b with Gensini score in CHD patients

**FIGURE 3:**
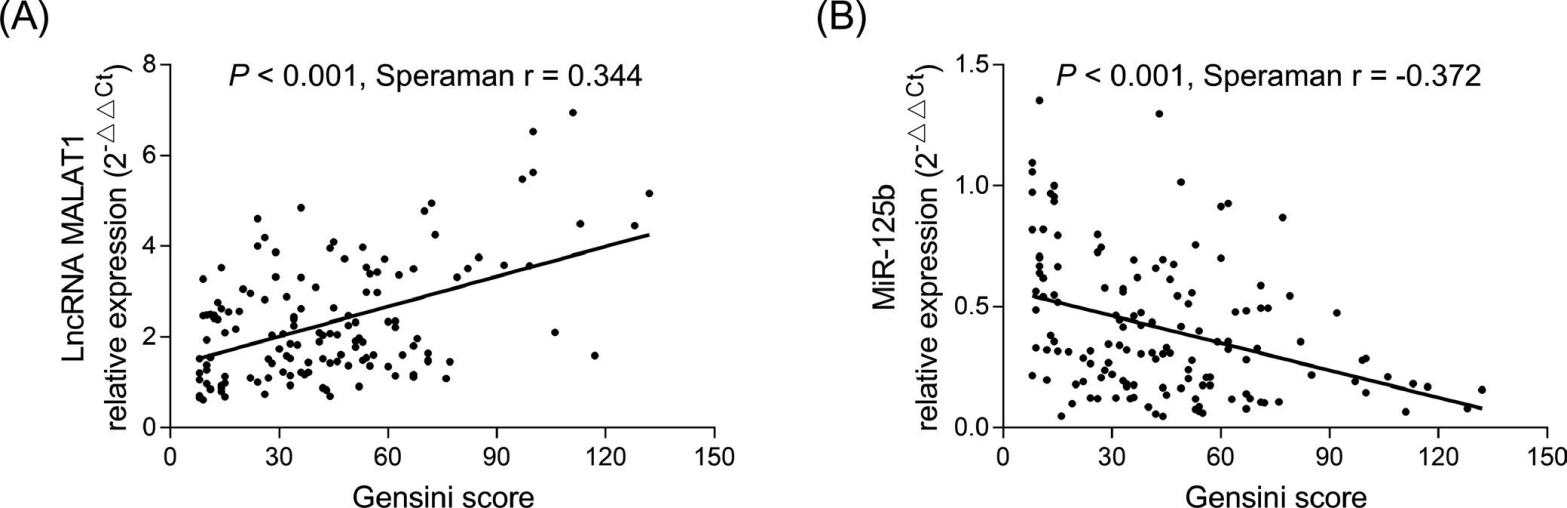
Correlation of lncRNA MALAT1 and miR‐125b with coronary stenosis severity in CHD patients. Correlation of lncRNA MALAT1 with Gensini score (A). Correlation of miR‐125b with Gensini score (B). CHD, coronary heart disease; lncRNA MALAT1, long non‐coding RNA metastasis‐associated lung adenocarcinoma transcript 1; miR‐125b, microRNA 125b

In CHD patients, lncRNA MALAT1 was positively associated with Gensini score (*r* = 0.344, *P* < .001) (Figure 3A); however, miR‐125b was negatively correlated with Gensini score (*r* = −0.372, *P* < .001) (Figure 3B), indicating that CHD patients with increased lncRNA MALAT1/reduced miR‐125b had higher level of coronary artery stenosis.

### 3.6 Correlation of lncRNA MALAT1 and miR‐125b with key biochemical indexes in CHD patients

In CHD patients, lncRNA MALAT1 was positively associated with TC (*r* = 0.216, *P* = .010), LDL‐C (*r* = 0.239, *P* = .005), and CRP (*r* = 0.359, *P* < .001). On the contrast, in CHD patients, miR‐125b was negatively correlated with TC (*r* = −0.177, *P* = .037), LDL‐C (*r* = −0.253, *P* = .003), and CRP (*r* = −0.355, *P* < .001), but positively associated with HDL‐C (*r* = 0.263, *P* = .002) (Table 3). More detailed information about correlation of lncRNA MALAT1 and miR‐125b with key biochemical indexes in CHD patients is listed in Table 3.

**TABLE 3:** Correlation of lncRNA MALAT1 and miR‐125b with major biochemical indexes in CHD patients

Items	lncRNA MALAT1		MiR‐125b	
	*P* value	Spearman's *r*	*P* value	Spearman's *r*
FBG	.212	.106	.996	.000
Scr	.791	.023	.076	−.150
SUA	.153	.121	.359	−.078
TG	.482	.060	.072	−.153
TC	.010	.216	.037	−.177
LDL‐C	.005	.239	.003	−.253
HDL‐C	.151	−.122	.002	.263
CRP	<.001	.359	<.001	−.355

Abbreviations: CHD, coronary heart disease; CRP, C‐reactive protein; FBG, fasting blood glucose; HDL‐C, high‐density lipoprotein cholesterol; LDL‐C, low‐density lipoprotein cholesterol; lncRNA MALAT1, long non‐coding RNA metastasis‐associated lung adenocarcinoma transcript 1; miR‐125b, mircroRNA‐125b; Scr, serum creatinine; SUA, serum uric acid; TC, total cholesterol; TG, triglyceride.

### 3.7 Correlation of lncRNA MALAT1 and miR‐125b with inflammatory cytokines in CHD patients

In CHD patients, lncRNA MALAT1 was positively associated with TNF‐α (*r* = 0.265, *P* = .002), IL‐1β (*r* = 0.236, *P* = .005), IL‐6 (*r* = 0.247, *P* = .003), and IL‐17 (*r* = 0.316, *P* < .001), but negatively correlated with IL‐10 (*r* = −0.305, *P* < .001) (Table 4). Meanwhile, miR‐125b was positively associated with IL‐10 (*r* = 0.226, *P* = .007), but negatively correlated with TNF‐α (*r* = −0.272, *P* = .001), IL‐6 (*r* = −0.205, *P* = .015), IL‐8 (*r* = −0.181, *P* = .033), and IL‐17 (*r* = −0.307, *P* < .001) (Table 4).

**TABLE 4:** Correlation of lncRNA MALAT1 and miR‐125b with inflammatory cytokines in CHD patients

Items	lncRNA MALAT1		MiR‐125b	
	*P* value	Spearman's *r*	*P* value	Spearman's *r*
TNF‐α	.002	.264	.001	−.272
IL‐1β	.005	.236	.117	−.133
IL‐6	.003	.247	.015	−.205
IL‐8	.057	.161	.033	−.181
IL‐10	<.001	−.305	.007	.226
IL‐17	<.001	.316	<.001	−.307

Abbreviations: CHD, coronary heart disease; IL, interleukin; lncRNA MALAT1, long non‐coding RNA metastasis‐associated lung adenocarcinoma transcript 1; miR‐125b, mircroRNA‐125b; TNF, tumor necrosis factor.

### 3.8 Correlation of lncRNA MALAT1 and miR‐125b with accumulating MACE occurrence in CHD patients

**FIGURE 4:**
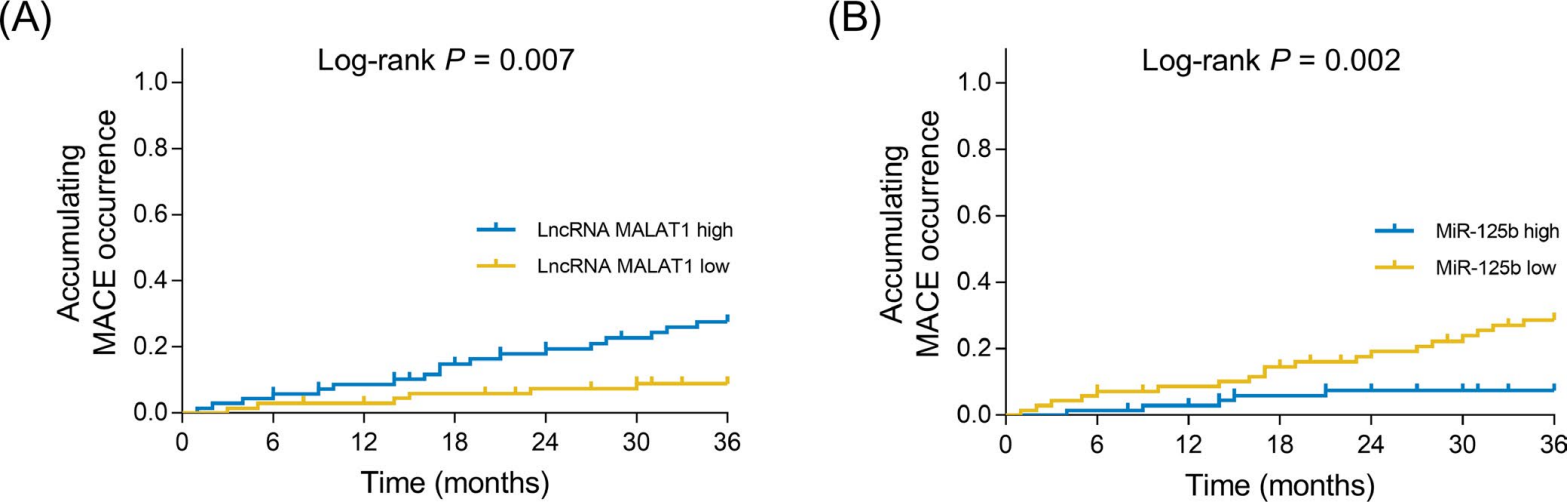
Correlation of lncRNA MALAT1 and miR‐125b with prognosis in CHD patients. Correlation of lncRNA MALAT1 with accumulating MACE occurrence (A). Correlation of miR‐125b with accumulating MACE occurrence (B). CHD, coronary heart disease; lncRNA MALAT1, Long non‐coding RNA metastasis‐associated lung adenocarcinoma transcript 1; miR‐125b, microRNA 125b; MACE, major adverse cardiovascular events

According to the median value of lncRNA MALAT1, patients were categorized as lncRNA MALAT1‐high patients and lncRNA MALAT1‐low patients, and lncRNA MALAT1‐high expression was associated with increased accumulating MACE occurrence (*P* = .007) (Figure 4A). Furthermore, based on the median value of miR‐125b, patients were divided into miR‐125b‐high patients and miR‐125b‐low patients, and miR‐125b‐high expression was correlated with reduced accumulating MACE occurrence (*P* = .002) (Figure 4B).

## 4 DISCUSSION

In the present study, we found that (a) lncRNA MALAT1 was increased in CHD patients compared with controls and positively associated with CHD risk independently. However, miR‐125b was reduced in CHD patients compared with controls and negatively correlated with CHD risk independently. Furthermore, lncRNA MALAT1 displayed a closely negative correlation with miR‐125b in CHD patients. (b) In CHD patients, increased lncRNA MALAT1 but decreased miR‐125b were correlated with higher severity of coronary stenosis, hyperlipidemia, and systematic inflammation. (c) In CHD patients, lncRNA MALAT1‐high expression but miR‐125b‐low expression was correlated with higher accumulating MACE occurrence.

Existing evidence demonstrates miR‐125b acts as endogenous sponge of lncRNA MALAT1, and lncRNA MALAT1 has regulatory effect in cardiac inflammation and dysfunction via targeting miR‐125b.^11^, ^12^ Furthermore, both lncRNA MALAT1 and miR‐125b are involved in the regulation of lipid metabolism disorder, vascular inflammation, and the pathogenetic implications of cardiovascular diseases (including atherosclerosis, vascular inflammation, and vascular calcification).^7^, ^8^, ^13^, ^14^ As far as we know, there is no clinical study yet regarding the association of lncRNA MALAT1 and miR‐125b with CHD risk, as well as their potential to predicting prognosis in CHD patients.

We conducted the present study, which observed that lncRNA MALAT1 expression was increased, while miR‐125b expression was decreased in CHD patients compared with controls. Furthermore, lncRNA MALAT1 was an independent risk factor for CHD, while miR‐125b was an independent protective factor for CHD. The possible reasons might include that (a) lncRNA MALAT1 might stimulate the upregulation of CRP, which was an important inflammatory factor involved in the formation of atherosclerosis; therefore, upregulation of lncRNA MALAT1 was independently associated with susceptibility of CHD.^20^ (b) Based on the prior evidence, miR‐125b in endothelial cells had inhibitory effect on endothelin‐1; therefore, decreased miR‐125b activated the secretion of endothelin‐1, which led to vasoconstrictive effect and increased the risk of hypertension, one of CHD risk factors, hence enhancing the occurrence of CHD. Meanwhile, decreased miR‐125b expression promoted the endothelial proliferation as well as migration, exacerbating the progression of atherogenesis and promoting the onset of CHD.^13^, ^20^ (c) Additionally, according to existing evidence that the pathological implications of CHD included chronic inflammation, we speculated that the progression of CHD might promote pro‐inflammatory cytokine‐induced lncRNA MALAT1 expression, but reduce p53‐mediated miR‐125b expression.^21^, ^22^ Meanwhile, there existed a closely negative correlation between lncRNA MALAT1 and miR‐125b in CHD patients. The possible reason might include as follows: lncRNA MALAT1 might activate several signaling pathways (such as p38 MAPK/nuclear factor‐kappa B (NF‐κB)) and interact with miR‐125b to induce cardiac dysfunction and promote the secretion of blood inflammation cytokinesis as in sepsis model, enhancing the generation of atherogenesis and further increasing the CHD risk.^4^, ^11^ These suggested that lncRNA MALAT1 might be an independent risk factor for CHD via interaction with miR‐125b. However, the negative correlation of lncRNA MALAT1 with miR‐125b was less obvious in controls, which might be explained by the fact that the difference of lncRNA MALAT1 with miR‐125b in controls was not as significant as in CHD patients.

Subsequently, we further observed that lncRNA MALAT1 was positively but miR‐125b was negatively associated with severity of coronary stenosis, hyperlipidemia, and systematic inflammation in CHD patients. The possible reasons might include that (a) according to the prior evidence, lncRNA MALAT1 was supposed to prevent the protective effect of miR‐125b against the development of CHD such as atherosclerosis, vascular inflammation, and vascular calcification, which led to higher severity of CHD.^12^, ^13^ (b) In addition, increased lncRNA MALAT1 mediated the release of neutrophil extracellular traps, further triggering hyperlipidemia in ox‐LDL‐treated atherosclerosis model. Meanwhile, miR‐125b was found to regulate scavenger receptor class B type I expression and present potential in the positive regulation of lipid metabolism disorder; therefore, lncRNA MALAT1 was positively correlated with hyperlipidemia, but miR‐125b was negatively associated with hyperlipidemia in CHD patients.^23^, ^24^ (c) Based on prior evidence, lncRNA MALAT1 might activate the several signaling pathway (such as NF‐κB pathway) via sponging miR‐125b, further leading to inflammation cascade. Hence, high lncRNA MALAT1 expression but low miR‐125b expression was correlated with increased pro‐inflammatory cytokines (CRP, TNF‐α, IL‐1β, IL‐6, IL‐17) and reduced anti‐inflammatory cytokines (IL‐10) in CHD patient; however, the detailed mechanism of lncRNA MALAT1 and miR‐125b involved in the CHD pathology needed to be explored by further cellular experiments.

Following that, we explored the potential of lncRNA MALAT1 and miR‐125b as indicators for MACE occurrence in CHD patients and found that lncRNA MALAT1‐high expression but miR‐125b‐low expression was associated with increased accumulating MACE occurrence in CHD patients. The possible reasons might include that according to the prior finding, increased lncRNA MALAT1 but decreased miR‐125b were associated with higher severity of coronary stenosis, hyperlipidemia, and systematic inflammation, which were common indicators for MACE occurrence, in CHD patients; hence, lncRNA MALAT1 and miR‐125b might be associated with MACE occurrence via interaction with these factors in CHD patients.

However, some limitations still existed in our study as follows: (a) Firstly, as 230 patients were from one single center, selection bias might exist, and more patients from multiple centers were needed to further verify the results in our study. (b) The follow‐up duration was 36 months in our study; thus, the predictive value of lncRNA MALAT1/miR‐125b for MACE incidence is needed to be investigated for a longer period in CHD patients. (c) The underlying mechanism of interaction between lncRNA MALAT1 and miR‐125b was not included in our clinical study, which could be further explored in the future.

In summary, both lncRNA MALAT1 and miR‐125b show potentials to be independent indicators for CHD risk and associate with disease severity and accumulating MACE occurrence in CHD patients, suggesting their possible clinical application as biomarkers in the CHD screening and surveillance.

## CONFLICT OF INTEREST

The authors declare that they have no conflicts of interest.
